# Exploring Musical Activities and Their Relationship to Emotional Well-Being in Elderly People across Europe: A Study Protocol

**DOI:** 10.3389/fpsyg.2017.00330

**Published:** 2017-03-20

**Authors:** Jennifer Grau-Sánchez, Meabh Foley, Renata Hlavová, Ilkka Muukkonen, Olatz Ojinaga-Alfageme, Andrijana Radukic, Melanie Spindler, Bodil Hundevad

**Affiliations:** ^1^Department of Cognition, Development and Educational Psychology, University of BarcelonaBarcelona, Spain; ^2^Cognition and Brain Plasticity Unit, Bellvitge Biomedical Research InstituteBarcelona, Spain; ^3^Department of Psychology, National University of IrelandGalway, Ireland; ^4^Department of Psychology, Masaryk UniversityBrno, Czechia; ^5^Department of Psychology, University of HelsinkiHelsinki, Finland; ^6^Department of Psychology, University of DeustoBilbao, Spain; ^7^Department of Psychology, University of Banja LukaBanja Luka, Bosnia and Herzegovina; ^8^Department of Psychology, University of OldenburgOldenburg, Germany; ^9^Department of Psychology, University of ViennaVienna, Austria

**Keywords:** elderly population, music, well-being, emotion, cross-cultural

## Abstract

Music is a powerful, pleasurable stimulus that can induce positive feelings and can therefore be used for emotional self-regulation. Musical activities such as listening to music, playing an instrument, singing or dancing are also an important source for social contact, promoting interaction and the sense of belonging with others. Recent evidence has suggested that after retirement, other functions of music, such as self-conceptual processing related to autobiographical memories, become more salient. However, few studies have addressed the meaningfulness of music in the elderly. This study aims to investigate elderly people’s habits and preferences related to music, study the role music plays in their everyday life, and explore the relationship between musical activities and emotional well-being across different countries of Europe. A survey will be administered to elderly people over the age of 65 from five different European countries (Bosnia and Herzegovina, Czechia, Germany, Ireland, and UK) and to a control group. Participants in both groups will be asked about basic sociodemographic information, habits and preferences in their participation in musical activities and emotional well-being. Overall, the aim of this study is to gain a deeper understanding of the role of music in the elderly from a psychological perspective. This advanced knowledge could help to develop therapeutic applications, such as musical recreational programs for healthy older people or elderly in residential care, which are better able to meet their emotional and social needs.

## Introduction

Many important changes such as retirement, changes in social ties and decline in physical and cognitive capabilities, occur during the later years of life. They can affect the psychological well-being of the elderly, and lead to loneliness (Luanaigh and Lawlor, 2008; Golden et al., 2009) and depression (Alexopoulos, 2005). Although defining well-being is challenging due to its multidimensional nature, there have been several attempts focusing mainly on the constructs rather than on the definition of well-being itself. In this regard, well-being can be understood as the balance point between an individual’s resources and challenges (Dodge et al., 2012). The presence of positive emotions and absence of negative ones as well as satisfaction with life and functioning are components of well-being (Diener et al., 2000; Ryff and Keyes, 1995). Several factors, such as personality and health, are known to correlate with well-being (Kahneman and Deaton, 2010). Moreover, studies addressing the role of leisure activities have found that they contribute to subjective well-being, and that the effect holds in cross-sectional, longitudinal, and experimental studies (Kuykendall et al., 2015). Specifically in the context of the elderly, participation in leisure activities seems to increase well-being and can reduce the risk of dementia (Herzog et al., 1998; Silverstein and Parker, 2002; Verghese et al., 2003; Adams et al., 2011; Chang et al., 2014).

Musical engagement, as part of everyday life, can positively influence and contribute to well-being. To evoke emotions, is one of the primary reasons for listening to or producing music (Juslin and Västfjäll, 2008; Croom, 2012; Koelsch, 2014). Cross-cultural research investigating the role of music on emotion suggests that emotional cues in music transcend both language and culture (Kim et al., 2010). Music can be used for emotional self-regulation through different strategies, such as diverting from contemplation over negative emotions, maintenance of positive mood, or relaxation; these strategies seem to be stable across the lifespan (Saarikallio, 2011). Musical activities are also an important source for social contact, promoting interaction and the sense of belonging with others (Rilling et al., 2002; Koelsch, 2014). Therefore, activities involving music such as listening to music, playing, singing, and dancing have been shown to have great impact on well-being, increasing the person’s life satisfaction.

To date, most research on the importance of music has focused on its cognitive and emotional functions, without consideration of the collective features of the musical experience. This is despite several researchers demonstrating the central importance of music to social and cultural settings (DeNora, 2000). Research has shown that the use of music for the self-regulation of mood is broadly similar across several disparate cultures, including Latin American, African, and European states (Saarikallio, 2008; Boer and Fischer, 2010). Some culture specific variations in the function of music were identified among sub-groups of these studies; this is in keeping with the wide scope of cultures examined. However, there have been no studies investigating the role of music in well-being in more similar cultures, such as between different European nations.

Despite the significant contribution of music to well-being, it is still not clear whether the importance of music remains stable across the lifespan (Lonsdale and North, 2011). Especially in senior years, musical activities can be accompanied by overcoming many barriers, such as loss of hearing, decline in memory, and an aging body. Musical importance in general is shown to be independent of level of mental competence and is also correlated with musical involvement (Cohen et al., 2002), however, no major differences between musicians and those without musical education have been found (Hays and Minichiello, 2005a). Compared to younger generations, a small decrease of importance of music in the elderly has been reported, one possible reason might be that the elderly simply do not have as many opportunities to listen to music they value as the younger generations do (Cohen et al., 2002; Laukka, 2007).

Nevertheless, the majority of elderly people listen to music on a daily basis (Cohen et al., 2002). Research indicates that the primary motive for engaging in activities involving music during the elderly is maintaining the identity and agency, and regulating mood (Laukka, 2007). Moreover, music helps the elderly understand their emotions, maintain their sense of well-being as well as to give them hope or meaning to live (Hays and Minichiello, 2005b). Music can be a source of relaxation and enjoyment (Laukka, 2007). Further, it can be a useful tool for expressing spirituality and can sometimes serve as an escape from everyday living through imagination or the evocation of memories (Hays and Minichiello, 2005b; Schäfer et al., 2012). Although these functions of music become more salient after retirement, only few studies have addressed the meaningfulness of music in the elderly.

The present study aims to (i) investigate elderly people’s habits and preferences related to music in a cross-cultural European sample, (ii) study the role music plays in their everyday life, (iii) and explore the relationship between music and emotional well-being. In relation to these aims, a survey will be administered to people over the age of 65 from five different European countries (Bosnia and Herzegovina, Czechia, Germany, Ireland, and UK) and to a control group. Participants will answer a survey about basic sociodemographic questions, habits and preferences in their participation in musical activities and a well-being questionnaire. The study will explore the daily use of music among elderly people, and we expect to find a positive correlation between frequency of participation in musical activities and emotional well-being regardless of the country of origin of the participants.

## Materials and Equipment

### Participants

A total of approximately 700 participants will be recruited for the present study, an elderly group from the age of 65 (*N* = 350) and a control group ranging from 20 to 30 years (*N* = 350). All participants will have normal or corrected to normal vision, since they will have to read a written survey. Besides, participants will be oriented in time and space at the time of test administration because they will have to answer questions about present and past behaviors. They will be asked which date and day of the week it is as well as the place where they will be completing the questionnaire (place, floor, city, and country). These basic questions will allow us to exclude those possible participants who might be confused or do not have a normal cognitive functioning. Participants with hearing deficits will be excluded because the nature of this study is to study music-related behaviors. Participants who currently live in nursing homes or other care facilities will be excluded as well.

The recruitment of participants will take place in Banja Luka (Bosnia and Herzegovina), Brno (Czechia), Meath (Ireland), Bremen and Munich (Germany), and London (UK). The groups will be matched in terms of gender, and similar sample sizes and age distributions will be collected in the different cities.

### Design

This study is a transversal, observational study with both descriptive and analytic purposes.

### Survey

The survey is divided into three different sections consisting of the Participant’s profile, Musical profile, and Well-being questionnaire, which assess sociodemographic aspects, musical activities, and emotional well-being, respectively, through questions and standardized questionnaires. The materials described below were selected for their relevance to the research questions.

The survey will be administered to the elderly group in paper–pencil format and using an online survey platform for the control group. Prior to the administration of the survey, participants will receive an information sheet stating the study procedure and will sign an informed consent.

### Participant’s Profile

At the beginning of the survey, participants will complete a questionnaire regarding their sociodemographic background, leisure activities, and social support, as well as a short personality screening.

#### Sociodemographic Background

Information about gender, age, marital status, living situation, education, occupation, financial situation, and religiousness will be enquired.

#### Leisure Activities

Twelve different leisure activities will be presented and participants will have to answer how often do they participate in these activities by rating the frequency on a 7-point scale from (1) *never* to (7) *daily*. Following responses are possible: listening to or playing music, internet use, watching tv or films, doing crosswords or playing board games, reading, playing sports, walking, doing art activities, such as painting or drawing, gardening, going out for meals or coffee, going out with friends or family, and going to social clubs. This information will allow controlling for other leisure activities influencing well-being.

#### Social Support

The 12-item Multidimensional Scale of Perceived Social Support (Zimet et al., 1988) will be used to assess social support. This scale comprises three subscales each assessing a different source of support: (a) family, (b) friends, and (c) significant other. There are 12 statements that participants have to rate using a 7-point scale ranging from (1) *very strongly disagree* to (7) *very strongly agree*. The scale and subscales are scored through calculating the mean as according to the manual. Previous studies have shown that a greater social support is positively correlated to well-being (Siedlecki et al., 2014). Therefore, this factor should be taken into account as a possible confounding variable in the present study. The 12-item Multidimensional Scale of Perceived Social Support shows good internal reliability as well as moderate construct validity (α = 0.88; Zimet et al., 1988).

#### Personality Screening

The Big Five Personality Inventory-10 (BFI-10) (Rammstedt and John, 2007), which measures the Big-Five dimensions of personality, will be administered. The inventory has two items per each of the five personality dimensions: Extraversion, Agreeableness, Conscientiousness, Emotional Stability, and Openness to Experience. Every item uses the same stem, “I see myself as: …”. Participants have to rate the items on a 5-point scale ranging from (1) *disagree strongly* to (7) *agree strongly.* The scales are scored according to the manual. The reliability of the BFI-10 is adequate (Overall mean: *r* = 0.75, Extraversion: *r* = 0.83, Agreeableness: *r* = 0.68, Conscientiousness: *r* = 0.43, Neuroticism: *r* = 0.36, Openness: *r* = 0.45; Rammstedt and John, 2007).

In total, the Participant’s profile will take approximately 15 min to complete.

### Musical Profile

Participants will answer questions about their education in music, the importance of music for them, habits related to musical activities, style preferences in music, and roles and functions of music in their everyday life.

#### Mastery

Participants will be asked whether they received special music-related education (such as playing an instrument, singing, and dancing classes).

#### Importance of Music

The first question assesses the subjective importance of music in the participant’s everyday life. Participants will be asked about how important music is for them with answers ranging from (1) *not at all* to (7) *extremely important.*

#### Habits

To assess the individual habits with regard to musical activities, participants will be asked about the frequency of doing different musical activities (listening to music, singing, playing an instrument, and dancing). A 7-point scale will be presented where they will have to rate how often do they do these activities from (1) *never* to (7) *daily*. Afterward, participants will be asked to estimate how long they listen to music on a normal day (in hours). In addition, information about which devices are used for listening to music will be collected. The possible responses for this last question are: radio, television, stereo, computer, portable device (i.e., walkman, discman, MP3 player), smartphone, concerts, car and public place.

#### Preferences

Nine different styles of music will be presented, these include: classical music; religious music; country, folk; jazz, swing, blues; disco, electronic; punk, rock, metal; hip-hop, rap; pop; and reggae, ska. Participants will be asked to judge their level of enjoyment [7-point scale from (1) *dislike extremely* to (7) *enjoy extremely*] with regard to these musical styles. In order to examine how content people feel about the accessibility of their preferred music, a 7-point scale from (1) *not at all* to (7) *very much* will be used.

#### Roles and Functions of Music

In order to gain insight on the roles and functions music plays in everyday life, participants will be asked to report the frequency of listening to music due to certain reasons. This question contains 24 reasons to listen to music (e.g., for entertainment, to stir up energy), which can be rated from (1) *very seldom* to (5) *very often*. This scale, which originates from a study conducted by Laukka (2007), assesses listening strategies, intending to cover main psychological functions of music listening (i.e., emotional functions, identity, belonging, and agency; e.g., Ruud, 1997).

The Barcelona Music Reward Questionnaire will be used to measure musical reward experience (Mas-Herrero et al., 2013). The Barcelona Music Reward Questionnaire decomposes music reward into five factors: Musical Seeking, Emotion Evocation, Mood Regulation, Social Reward, and Sensory-Motor. Musical reward experience is measured using 20 statements that participants are asked to rate from (1) *completely disagree* to (5) *completely agree.* Scoring is done according to the manual, resulting in a score for each facet and a score for the global sensitivity for music reward. The Barcelona Music Reward Questionnaire also presents good reliability estimates (*r* = 0.92; Mas-Herrero et al., 2013).

To assess different emotions experienced with music, participants will report the frequency of different emotions they felt in response to listening to music, playing an instrument, singing and dancing. Participants are asked to rate 7 different emotions (e.g., happy, nostalgic, moved) on a scale from (1) *never* to (7) *always*. The original question was reported in Laukka (2007), and consisted of 45 emotions. In order to shorten the time of administration for the present study, these were summarized into more basic emotions, which are: happy, nostalgic, anxious, moved, bored, frustrated, sad, lonely, thrills or chills, disappointed, tense, angry, spiritual and relaxed.

The musical profile will take approximately 20 min.

### Well-Being Questionnaire

At the beginning of this part of the questionnaire, participants will be asked whether they experience hearing problems, to control for this possible influence on musical experiences. In order to assess emotional well-being, different validated scales will be administered measuring emotional state, health status, and quality of life.

#### Emotional State

The Positive and Negative Affect Scale, which is comprised by two mood scales: the Positive Affect Scale and the Negative Affect Scale (Watson et al., 1988), will be administered. Ten descriptors are used to define each scale and participants will be asked to respond whether they have felt these emotions in the last week using a 5-point scale that ranges from (1) *very slightly or not at all* to (5) *extremely.* The Positive and Negative Affect Scale has strong reported validity with measures as general distress and dysfunction, depression, and state anxiety. It was also successfully validated for the elderly population (PA: α = 0.87, NA: α = 0.89; Kercher, 1992).

#### Emotion Regulation

To assess emotion regulation problems, the short form of the Difficulties in Emotion Regulation Scale (Kaufmann et al., 2015) will be administered, which consists of 18 items, with answers ranging from (1) *almost never* to (5) *almost always*. The items are grouped into six scales: Strategies, Non-acceptance, Impulse, Goals, Awareness, and Clarity. The short form of the Difficulties in Emotion Regulation Scale will be scored according to the manual and maintains good psychometric properties (α ≥ 0.70; Kaufmann et al., 2015).

#### Resilience

The 10-item Connor–Davidson Resilience Scale (Connor and Davidson, 2003) will be administered in order to assess resilience, by focusing on personal resources or qualities deemed appropriate for positive adaptation to adversity. The scale is comprised of 10 items, each rated on a 5-point scale from 0 to 4, with higher scores reflecting greater resilience. The psychometric properties are good (α = 0.85*;* Campbell-Sills and Stein, 2007).

#### Health Status

Selected subscales from the Short Form Health Survey (Ware and Sherbourne, 1992) will be used to measure emotional role functioning, emotional well-being, and general health. The Short Form Health Survey also showed good reliability and construct validity in terms of distinguishing between groups with expected health differences (α ≥ 0.85*; r* ≥ 0.75; Brazier et al., 1992).

#### Subjective Well-Being

The Satisfaction with Life Scale (Diener et al., 1985) will be administered to screen subjective well-being. It consists of five items asking about satisfaction with one’s life on a 7-point scale from (1) *strongly disagree* to (7) *strongly agree*.

The Well-being questionnaire will take around 30 min to complete, resulting in a total administration time of 65 min.

## Stepwise Procedures

### Ethics

The study is approved by the ethical board of the University of Barcelona. Each participant will receive information about the aim and procedures of the study and will provide consent for participation.

### Translations of the Survey

Since participants will be recruited from different countries, translated versions of the survey are needed for those countries in which English is not the official language. Therefore, the survey has been translated to Czech, German, and Serbo-Croat-Bosnian following a forward and back translation process for questions and standardized questionnaires that were not available in those languages. First, a native speaker of the target language with a proficiency level in English translated the original version of the survey in English to the target language (forward translation). Following the guidelines of the WHO for instrument translation, the local researcher of the study provided instructions to the translator, aiming for conceptual rather than literal translations. Second, an expert panel reviewed the forward translation, identifying and discussing inadequate expressions until reaching consensus (reconciliation). The expert panel was composed by three psychologists that were native speakers of the target language with a proficiency level in English and the local researcher. Third, an English native speaker with a proficiency level in the target language translated the survey from the target language to English (back translation). As in the forward translation, the local researcher provided guidelines for a conceptual translation. At the end of the process, the expert panel reviewed and compared both translations. If disagreements occurred in one word or expression, it was discussed until reaching a consensus (harmonization).

### Pilot Testing and Feasibility of the Survey

After translating the survey to the target languages, 45 pilots were conducted. The local researchers asked for feedback regarding the clarity and length of the survey, the presentation of the questions and statements, and the display of the items.

### Recruitment of Participants

For each recruitment location, a list of social clubs, civil societies, and neighborhood associations will be created with information about the nature of the club or society. Local researchers will contact the person in charge to introduce themselves, explain the purpose of the study and ask if there are members above 65 years old. If so, researchers will ask for permission to recruit participants and administer the survey in their venue. When visiting the clubs and societies, local researchers will explain the aim of the study to participants and ask for the individual consent of each participant. However, elderly participants from these clubs will be individuals relatively independent and active, and thus may not be a representative sample of the elderly people in general. To compensate this bias, local adult day-care centers will be approached following the same procedure as with social clubs and societies to target those individuals who are less active or involved in the community.

Regarding the control group, local researchers will post announcements about the study in different social networks, web pages and online forums. To target the control population (from 20 to 30 years old), local researchers will take into account the nature and content of these online sources. For both groups, the elderly and the control group, the aim of the study will not be disclosed in advance to prevent from attracting participants with a special interest in music.

### Administration of the Survey

A guideline for local researchers will be used to explain the nature of the study to participants, and to provide instructions and guidance for each of the questions of the survey should the participant required assistance. The elderly group will complete the survey in a quiet environment in either single- or small group-sessions whereas the control group will answer using an online platform for surveys.

## Proposed Analysis

The data collected in different recruitment locations will be managed by each local researcher, who will be responsible for gathering the data in a template database. Statistical analysis will be performed using the Statistical Package for the Social Sciences (IBM SPSS Statistics 23, Armonk, NY, USA). Since the data will be collected in different countries, we will test for measurement invariance before performing any analysis.

For the first aim of the study, which is to investigate the habits and preferences related to music in the elderly in a cross-cultural European sample, descriptive analyses will be performed for variables obtained from the music profile of the survey. These variables are the (1) importance of music, (2) frequency of different musical activities, (3) length and devices for listening to music, (4) music style preferences and level of enjoyment, (5) access to the preferred music, (6) reward and (7) emotions associated to music. These descriptive analyses will be performed first separately per each country (Bosnia and Herzegovina, Czechia, Germany, Ireland, and UK). Exploratory analyses comparing the results of the elderly participants and the control group of each country will be performed using independent *t*-tests. We will also apply descriptive statistics with all the data gathered from the different countries and the results of the overall elderly sample will be compared to the control group to test for differences between age groups.

The same type of analysis, descriptive and exploratory, will be applied to the second aim, which is to study the role of music in everyday life, by selecting the responses obtained from the question about the reasons for engaging in different musical activities. Analysis will be performed first separately for each country and comparing with the control group and then with all the subsamples together. Two-tailed significance tests will be used.

With regard to the third aim, exploring the associations between participation in musical activities (listening to music, singing, playing and instrument and dancing) and emotional well-being, a hierarchical multiple regression with two steps will be applied. Our hypothesis is that participants with higher frequency of participation in musical activities will report higher emotional well-being regardless of the country of origin of the participants.

On the first step of the regression, a model will be created with the information of the Participant’s profile and Well-being questionnaire of the survey. The sociodemographic data will be the independent predictor variables, namely: (1) age, (2) marital status, (3) living situation, (4) education, (5) occupation, (6) religiousness, (7) participation in leisure activities, (8) perceived social support, and (9) personality. For the dependent variables, the Satisfaction With Life Scale is the one which is mostly hypothesized to be affected. However, we will use other scales measuring wellbeing (the Positive and Negative Affect Scale, the Difficulties in Emotion Regulation Scale, the Connor–Davidson Scale, and the selected questions from the Short Form Health Survey) as there is no clear evidence on which practicing music would affect most. We will test them all with the appropriate family-wise error corrections. The variable country will be introduced as a fixed effect. With this step, we expect to have a clearly significant model since previous research supports the idea that some demographic aspects (e.g., marital status, or education or socioeconomic status) have a positive influence on the emotional well-being of individuals (Kahneman and Deaton, 2010). That is why these variables will be included into the model as control variables. Based on previous research on different factors on well-being (Laukka, 2007; Boarini et al., 2012; Oguz et al., 2013), we estimated our background factors to explain *r*^2^ = 0.35 of the variance of different well-being measures.

On the second step, the variable about frequency of musical activities will be introduced in the model. With this step, we will test whether the inclusion of participation in musical activities improves the model of well-being. With our estimated sample size (350 elderly participants), we can expect to have >0.95 power with our independent measure adding at least 0.02 to the *R*^2^ of the whole model, after correcting for multiple testing (Bonferroni) for our five different well-being measures. The same procedure will be applied with the participants in the control group and the possible differences between groups will be examined.

## Anticipated Results

The first aim of this study is to investigate elderly people’s habits and preferences related to music. Based on previous studies, we expect to find that while most elderly people from our sample will be daily listeners, the amount of singers, instrument players or dancers will be lower (Cohen et al., 2002). Moreover, we expect to find a lower frequency of musical activities among elderly people when compared to the control group (Cohen et al., 2002). The main reason for this could be that elderly people do not have as many opportunities to engage in musical activities as younger people do (Cohen et al., 2002; Laukka, 2007). It is thought that music styles and preferences are established during adolescence and early adulthood (Cohen et al., 2002). Indeed previous research indicates that elderly persons have a strong preference for music popular during their youth (Gibbons, 1977). However, there is little recent research investigating this topic, and none of a cross-cultural nature. Nonetheless, we expect to find that both the elderly and control group will show a preference for music popular in their youth.

The second aim of the study is to examine the role music plays in elderly people’s everyday life. We anticipate that the importance of music in elderly persons will be evident in its utilization for purposes such as maintaining well-being, entertainment, self-identity, or socializing (Hays and Minichiello, 2005a,b). Furthermore, we expect to find some differences between the elderly and the control group. For example, in the role of music for mood management or emotional regulation, being this more prevalent in younger persons, or functions such as self-conceptual processing related to autobiographical memories being more salient in the elderly (Lonsdale and North, 2011). Nevertheless, we expect music to have the same importance in both groups, independent of age, mental competence and region (Cohen et al., 2002), and for it to be either more important or more frequent than other leisure activities for a large amount of participants (Lonsdale and North, 2011).

Based on previous research, we expect to have a significant model of background personal variables, such as religiousness, marital status, gender, social support, and personality influencing emotional well-being (Lee and Ishii-Kuntz, 1987; Kahneman and Deaton, 2010). This model will help us to control for possible confounding variables when assessing the relationship between music and well-being. Introducing the frequency of musical activities in this model, we expect that a higher involvement in musical activities will be related to a better emotional well-being, in both, the elderly and the younger group. We expect to find that participation in musical activities, and in particular group activities, will increase emotional well-being, largely as a result of an increase in positive affect, social contact, shared experience, and evocation of positive memories (Laukka, 2007; MacDonald, 2013).

No differences between participants with and without professional musical background are expected (Hays and Minichiello, 2005a). However, due to increased musical education in recent years, we anticipate that formal musical education may be greater in the control group. In accordance with previous literature, we expect that there will be no significant cultural differences in the role of music in emotional well-being (Argstatter, 2016). Studies about neuroscience of music have provided explanation for a similar global experience of music, identifying that music activates the reward, emotion, and arousal regions of the brain (Blood and Zatorre, 2001). This could explain why an increase in positive affect and positive emotion related to musical activities appears to transcend both culture and region.

Despite every effort to control for confounding variables, this study will have a number of limitations. Our strategy for recruitment will lead to a convenient sample of social clubs and churches will likely lead to an over-representation of active elderly persons. Although, we intend on preliminarily screening for participants with dementia, we do not have the resources to carry out full cognitive, neurological or psychiatric screening on participants involved in this study. However, as participants will all be active in the community it is likely that the effects of such conditions will be minimal.

The use of a cross-cultural sample is a limitation, in that individual cultural factors may influence our findings. Indeed, research does suggest that the function of music varies between cultures (Cross, 2001). However, it is essential to note that the focus of this research has to date been between vastly different cultures, such as western and south east Asian nations (Cross, 2001; Kim et al., 2010). In contrast, our study is confined to European countries with more similar musical traditions. Further, our investigation into musical habits and preferences will control for cultural differences in frequency and type of musical engagement.

The use of a number of short self-report questionnaires, may also lead to a several limitations. Although self-report questionnaires have been accused of being especially prone to participant bias in individual studies (Adams et al., 1999; Donaldson and Grant-Vallone, 2002), Chan’s (2009) chapter reviewing the literature finds that, although inflation of the observed correlation is a possibility in self-report literature it is not a necessity. While more comprehensive questionnaires often have superior psychometric properties, we have chosen to use shorter questionnaires, in order to prevent extra burden on the participant. This is especially important for an elderly population who may experience fatigue related decline at the end of long testing. For this reason, we have also declined a more in depth examination of the role of other leisure activities on emotional well-being in the elderly. However, this is one possible avenue for future research in the area. A limitation when collecting data in different countries is that we might need to exclude some of the scales or items in the analyses if they do not converge when testing for measurement invariance. Moreover, the use of different methods for data collection represents a limitation in this study when comparing groups. Finally, the use of a cross-sectional, correlational design will limit our ability to make causal interpretations from our research.

We expect our project to contribute to knowledge about music preferences and daily habits in elderly people across Europe. Moreover, this knowledge will help to develop a greater understanding of how music relates to emotional well-being in elderly people, and will therefore be useful to better design musical or recreational programs, not only for elderly persons who experience challenges associated with aging, but also for healthy elderly people. Especially during aging, musical activities can help to maintain physical and mental health and cognitive abilities, however, more research needs to be done to understand how the activities are connected with an individual to better design music interventions.

Music therapy has demonstrated its efficacy in a number of circumstances including dementia (Chu et al., 2014), cognitive decline (Mammarella et al., 2007), pain relief (McCaffrey and Freeman, 2003), and depression (Chu et al., 2014). Moreover, music therapy treatment improves global and social functioning in schizophrenia and serious mental disorders, Parkinson’s disease and sleep quality (Kamioka et al., 2014). There is a considerable body of research which indicates that music therapy interventions are most effective when “preferred music” is used, in comparison to relaxing or other music (Sung and Chang, 2005; Mitchell and MacDonald, 2006). A study illustrated the effectiveness of individualized music designed music interventions for two groups of elderly persons with Alzheimer’s disease and related disorders (Gerdner, 2000). One received individualized music and classical relaxation music, the other group received the same protocol in reverse order. Reduction in agitation was shown only during and following individualized music compared to classical relaxation music (Gerdner, 2000). However, elderly people might not be comfortable being involved in some music therapy interventions, as depicted the study by Burns et al. (2005). They suggest that people might be interested in music therapy involving music listening, but not music-making.

## Conclusion

Individual needs have to be taken into account while designing such interventions. Therefore, by using an exploratory method to identify musical preferences and the role of music in the elderly, we hope to contribute to more effective and targeted music therapy interventions, and to further the use of music to enhance emotional well-being in the elderly.

## Author Contributions

This study was conceived and initially designed by JG-S. All authors contributed equally to the research design and to the preparation of the manuscript.

## Conflict of Interest Statement

The authors declare that the research was conducted in the absence of any commercial or financial relationships that could be construed as a potential conflict of interest.
